# Abnormal expression of LCA and CD43 in SCLC: a rare case report and brief literature review

**DOI:** 10.1186/s12890-024-03005-w

**Published:** 2024-04-22

**Authors:** Zhe Cai, Linwei Zuo, Fangfang Hu, Huiyan You, Xiangtong Lu, Shousheng Liao, Fanrong Liu, Lixiang Li, Wenyong Huang

**Affiliations:** Department of Pathology, The Second Affiliated Hospital of Nanchang University, No. 1 Minde Road, Donghu District, 330000 Nanchang, China

**Keywords:** LCA, CD43, SCLC, Immunohistochemistry, Diagnostic pitfall

## Abstract

**Background:**

To present an unusual case of abnormal LCA expression and CD43 in SCLC and to review the reported literature to avoid potential diagnostic pitfalls.

**Case presentation:**

A 73-year-old male patient suffered from persistent back pain for more than one month. MRI revealed a compression fracture of the L1-L5 vertebra. A CT scan revealed multiple nodules and masses at the left root of the neck, lung hilum and mediastinum, and multiple areas of bony destruction of the ribs. Histology of the tumor revealed that small and round cells were arranged in nests with areas of necrosis. The tumor cells were round to ovoid with scant cytoplasm and indistinct cell borders. The nuclear chromatin was finely granular, and the nucleoli were absent or inconspicuous. Immunohistochemically, the tumor cells were positive for cytokeratin, TTF-1, POU2F3, LCA, and CD43.

**Conclusion:**

This report highlights a potential diagnostic pitfall in the diagnosis of SCLC, urges pathologists to exercise caution in cases of LCA and CD43 positivity and illustrates the need for further immunohistochemical studies to avoid misdiagnosis.

## Background

CD45 is a protein tyrosine phosphatase, also known as leukocyte common antigen (LCA), that is essential for the initiation of T-cell receptor signalling and is commonly used to diagnose lymphoid diseases [1].Although abnormal LCA expression has also been reported in nonhematopoietic malignancies, such as poorly differentiated colorectal adenocarcinoma, aggressive pituitary adenoma, spermatogonium, rhabdomyosarcoma, and metastatic undifferentiated carcinoma [2, 3], only three cases of lung neuroendocrine tumors with positive LCA expression have been reported in the literature [2, 4].

CD43 is a transmembrane sialoglycoprotein produced by leukemia cells that can regulate cell adhesion, signal transduction, apoptosis, migration and proliferation. CD43 is normally expressed on the membrane of lymphocytes and is a specific marker for tumors of the lymphohematopoietic system. Research has shown that some nonhematopoietic malignancies, such as colorectal cancer and adenoid cystic carcinoma, can also express CD43. However, all of these cells are weakly positive for CD43 in the nucleus of the tumor cells [5], and uniform strong membrane-positive expression of CD43 in nonhematopoietic malignancies has never been reported till now.

This paper presents an extremely rare case of abnormal expression of LCA and CD43 in small-cell lung cancer (SCLC) patients, aiming to improve the understanding of abnormal LCA and CD43 expression in SCLC, avoid diagnostic pitfalls, and provide case references for further research.

## Case presentation

**Fig. 1:**
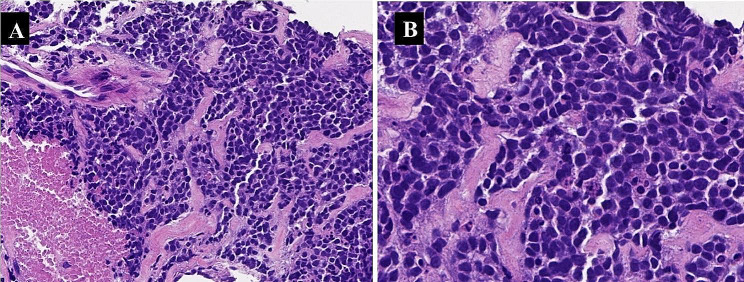
The tumor cells in this patient were arranged in sheets with nesting in places and areas of necrosis (H&E,×200)**(A)**. The tumor cells were round to ovoid with sparse cytoplasm and indistinct cell borders. Nuclear chromatin is finely granular and nucleoli are absent or inconspicuous (H&E,×400) **(B)**

**Fig. 2:**
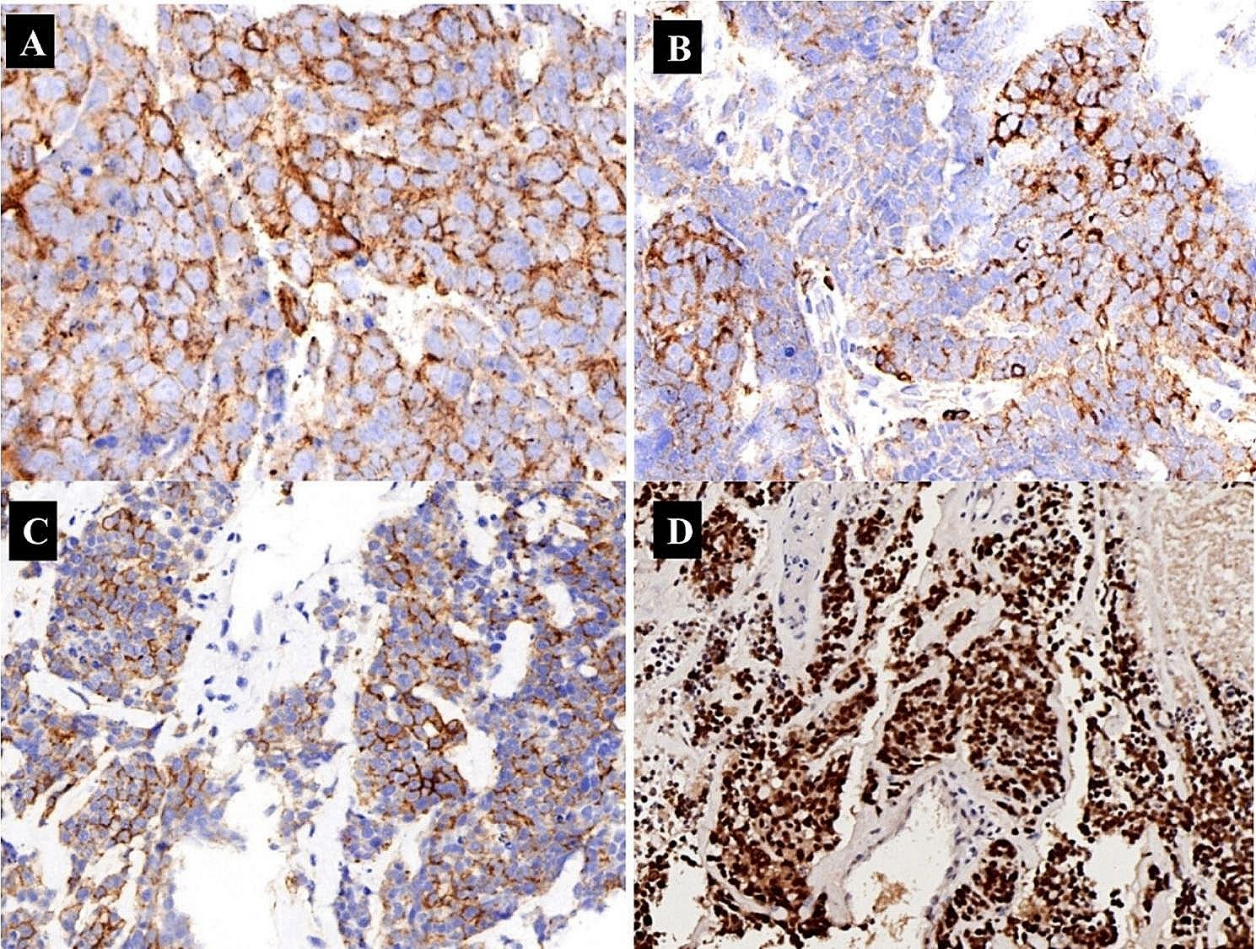
Immunohistochemically, the tumor cells showed strong punctate staining for cytokeratin **(A)**, patchy but strong membranous reactivity for LCA **(B)** and CD43 **(C)**, and strong nucleus staining for POU2F3 **(D)**

A 73-year-old male patient presented one month prior with persistent back pain and activity limitations. Magnetic resonance imaging (MRI) revealed compression fractures of the L1-L5 lumbar spine with spinal edema. A CT scan revealed multiple nodules and masses at the left root of the neck, lung hilum, mediastinum, and multiple bony destructions of the ribs. The histologic appearance was that of a small blue round cell tumor. The tumor cells were arranged in sheets with nesting in places and areas of necrosis (Fig. 1A). The tumor cells were round to ovoid with sparse cytoplasm and indistinct cell borders. Nuclear chromatin was finely granular and nucleoli were absent or inconspicuous (Fig. 1B). Immunohistochemically, the tumor cells showed strong punctate staining for cytokeratin (Fig. 2A), patchy but strong membranous reactivity for LCA (Fig. 2B) and CD43 (Fig. 2C), and positivity for TTF-1 and P63, and CD117 (KIT). Classical neuroendocrine markers (chromogranin, synaptophysin, and CD56 ) were negative in this case, and INSM1 was focally weakly positive, whereas nuclear staining for POU2F3 was strongly positive. (Fig. 2D). The patient had multiple tumor metastases at the time of diagnosis, and the treatment effect was inferior. Unfortunately, the patient died at the 3-month follow-up after diagnosis.

## Discussion

The expression of LCA and CD43 in nonhematopoietic malignancies has been rarely reported; here, we report a case of SCLC co-expressing LCA and CD43. In our patient, the tumor cells were positive for membrane expression of LCA and CD43. In addition, this patient showed strong positive expression of cytokeratin and POU2F3, weak focal positivity for INSM1, and negative expression of CgA, Syn, and CD56. Histologically, the tumor cells were arranged in sheets with nesting in places and areas of necrosis, showing strong punctate staining for cytokeratin, which is helpful in diagnosing SCLC, and were also positive for TTF-1 and POU2F3. Based on the morphology and immunohistochemical staining of the tumor cells, we excluded the possibility of lymphoma and finally made the diagnosis of SCLC.

LCA is expressed on nearly all hematolymphoid cells except erythroid cells and megakaryocytes, and at particularly high levels on lymphoid cells. Immunoreactivity in the LCA is highly suggestive of lymphoid malignancy. However, LCA can also be rarely expressed in nonhematopoietic malignancies such as various carcinomas and sarcomas [2, 3]. The reported cases of positive LCA expression in nonhematopoietic malignancies were mostly cytoplasmic and nuclear, and there were only three cases of lung neuroendocrine tumors showed the membrane-positive expression of LCA have been reported in the literature [2, 5]. In addition, membranous LCA positivity has also been reported on the surface of necrotic carcinoma cells [6], and overaggregated nuclei and cytoplasmic remnants result from artifactual distortion of hematopoietic elements in small tissue biopsies [7]. However, in our case, the LCA-positive cells were neither necrotic nor were they crushed aggregated cells to a significant degree.

Although CD43 has been widely used in the diagnosis of hematolymphoid neoplasms, a number of non-lymphoid tumors have been reported in the literature to occasionally show immunoreactivity for this marker. These included colorectal cancer and adenoid cystadenocarcinoma, all of which were weakly positive in the nucleus of the tumor cells but not on the membrane. A study on primary lung tumors showed that the expression of CD43 in the cytoplasm and nucleus of tumor cells, which can reduce intercellular adhesion, inhibit apoptosis and promote chemotherapy resistance, thereby promoting tumor progression [8].

Our study is the first to report membranous CD43 expression in SCLC. Seethala et al. have reported that some non-hematopoietic malignancies can also express CD43, but all of them are weakly positive in the nucleus of the tumor cells [5]. Careful interpretation of immunohistochemical staining is essential, especially when unexpected results are encountered. Therefore, it is important to interpret immunophenotyping in the appropriate morphologic context.

Cytokeratin was positive in our patient, with a punctate expression pattern. Although some lymphomas, especially when extranodal lymphomas, occasionally show cytokeratin positivity, while all cases reported in the literature are large cell anaplastic lymphomas [9, 10]. In addition to cytokeratin, POU2F3 also showed strong positive expression in our patient. POU2F3 is a transcriptional regulatory factor of cluster cells, and is mainly distributed in the gastrointestinal tract and bronchi [11]. Previously, the expression of POU2F3 was observed only in 15∼20% of SCLC cases, 70% of thymic squamous cell carcinoma cases and castration-resistant prostate adenocarcinoma (CRPC) cases [12]. SCLC with positive POU2F3 expression is belong to the chemically insensitive type, while SCLC typically does not express or has low expression of classic neuroendocrine markers such as CD56, chromogranin A, and synaptophysin [13], which is consistent with our case. Studies on the origin of POU2F3-positive SCLC indicate that POU2F3 expression cannot be proven to originate from cluster cells of the bronchial epithelium. It may be acquired by a gene trans-differentiation mechanism or genetic alteration [14]. Therefore, based on the morphology and location of tumor cells, the strong positive expression of POU2F3 in tumor cells may be a specific marker for SCLC.

Currently, SCLC is divided into four subtypes: the ASCL1-high (SCLC-A), NEUROD1-high (SCLC-N), POU2F3-high (SCLC-P) and YAP1-high (SCLC-Y) subtypes, which are enriched in WT RB1 [15]. The SCLC-P subtype of SCLC is mutually exclusive to ASCL1 and NEUROD1, and ASCL1/NEUROD1 double-negative SCLC represents a distinct neuroendocrine-low subtype [16–18]. In addition, studies have shown that patients with high expression of POU2F3 have a better response to chemotherapy with lurbinectedin and may have a significantly improved prognosis [19].

However, the biological role of LCA and CD43 expression in SCLC remains unclear. In our study, the patient had multiple systemic metastases at initial diagnosis and poor clinical signs during hospitalization, and died three months later. The significance of the positive expression of LCA and CD43 in the membrane of nonhematopoietic malignancies remains to be investigated. Whether this difference is related to poor tumor differentiation or poor patient prognosis requires more case support or experimental support at the molecular level.

SCLC patients with abnormal expression, including our patient. The two patients died three months and twelve months after discharge, respectively.

In conclusion, this article describes a patient with abnormal LCA and CD43 expression in SCLC, which highlights a potential diagnostic pitfall in the diagnosis of SCLC and illustrates the need for an immunohistochemical marker panel to avoid misdiagnosis.

## Data Availability

The data and materials are available upon request from the corresponding author.
